# Effects of major analgesics on male fertility: A systematic literature review

**DOI:** 10.5935/1518-0557.20240020

**Published:** 2024

**Authors:** Melissa Figueiredo Capelo, Paula Bruno Monteiro, Beatriz Matos Anastácio

**Affiliations:** 1 Christus University Center (UNICHRISTUS), Biomedicine Department. Fortaleza-Ceará, Brazil

**Keywords:** male infertility, hormonal changes, NSAIDs, sperm quality, human reproduction

## Abstract

**Objective:**

To verify, based on a systematic literature review, the effects of the main analgesics on male fertility.

**Data Sources:**

The studies were analyzed from the PubMed, SciELO and LILACS databases.

**Study Selection:**

The articles selected for the present review included: cohort studies; cross-sectional studies, clinical trials; complete studies; studies with animal models that addressed the proposed theme and that were published within the stipulated period from March 1, 2013, to March 31, 2023, in English, Portuguese and Spanish. These would later have to go through inclusion stages such as framing the type of study and exclusion criteria.

**Data Collection:**

Author’s name, year of publication, study population, number of patients, analgesic, administration time, dose, and effect.

**Conclusions:**

There are in vitro and in vivo studies that link paracetamol and ibuprofen to endocrine and seminal changes that are harmful to male fertility. However, more clinical research is needed to determine the doses and timing of administration that affect fertility. The effects of aspirin on male fertility are still unclear due to the lack of studies and consistent methodologies. There is not enough research on dipyrone and its relationship with male fertility, requiring more studies in this area.

## INTRODUCTION

Analgesics, broadly classified as opioids and non-opioids, encompass a diverse group of pain-relieving medications. The non-opioid category is notably heterogeneous, comprising subgroups such as non-steroidal anti-inflammatory drugs (NSAIDs), antipyretics, and other analgesics (Schneider *et al*., 2019). Due to its diverse nature, some medications within this class, despite long-standing use, have not had their mechanism of action and side effects completely elucidated, as is the case with paracetamol (PCM) (Castel-Branco *et al*., 2013). Nevertheless, this class of medications represents the most widely consumed globally, given its application in the management of mild to moderate pain and its availability without a prescription (McCrae *et al*., 2018).

In Brazil, analgesics stand as the most commonly used medications, as indicated by a study conducted by Borges *et al*. (2023). Among these, paracetamol, dipyrone, and aspirin rank as the most frequently utilized. Although ibuprofen was not explicitly mentioned in this category, it was grouped with anti-inflammatories like naproxen and diclofenac sodium. PCM, owing to its analgesic and antipyretic effects, enjoys widespread use on a global scale (Ennis *et al*., 2016). Its consumption is advocated for the treatment of acute and chronic pain, as well as for fever management. Furthermore, its use has been proposed in the closure of the ductus arteriosus in premature infants (Bührer *et al*., 2021). Its pharmacological properties mirror those of NSAIDs, suppressing prostaglandin production through the inhibition of cyclooxygenase (COX) enzymes, namely COX-1 and COX-2 (Jóźwiak-Bebenista & Nowak, 2014; Przybyła *et al*., 2021).

Dipyrone, also known as metamizole, stands as the most widely used analgesic in Brazil and is available over the counter in various forms. This medication possesses antipyretic, antispasmodic, analgesic, and anti-inflammatory properties (Knappmann & Melo, 2010). However, it is important to note that dipyrone can induce adverse effects, most notably agranulocytosis, leading to its discontinuation in several countries, including the United States, Canada, Denmark, and the United Kingdom (de Leeuw *et al*., 2017; Nikolova *et al*., 2012; Siramshetty *et al*., 2016). Although its mechanism of action has been extensively studied, it remains not fully understood. It is established that the metabolites 4-N-methylaminoantipyrine and amino antipyrine inhibit prostaglandin production, and its analgesic effect stems from the inhibition of COX enzymes, primarily COX-2 (Pereira, 2014; Rogosch *et al*., 2012).

One of the earliest synthesized drugs was aspirin (AAS), a notable representative of non-steroidal anti-inflammatory drugs (NSAIDs), and since its inception, it has held a prominent position as one of the most widely used analgesics. Moreover, it remains unparalleled as the most prevalent drug in the modern era (Fijałkowski *et al*., 2022). Its continued global consumption can be attributed to its diverse applications in the medical field, encompassing analgesic and antipyretic properties (Fuster & Sweeny, 2011). However, its antiplatelet characteristic is a key driver of its extensive use for preventing cardiovascular and cerebrovascular diseases (Montinari *et al*., 2019). The primary mechanism of action of AAS involves irreversible inhibition of COX-1, COX-2, and COX-3, achieved through the inhibition of prostaglandin precursors and thromboxane production (Sankaranarayanan *et al*., 2020; Ornelas *et al*., 2017).

Another crucial medication is Ibuprofen, belonging to the NSAIDs group and widely available in pharmacies worldwide. Primarily administered orally, it exhibits antipyretic, anti-inflammatory, and analgesic properties (Irvine *et al*., 2018). As commonly known, NSAIDs act through the inhibition of COX enzymes that metabolize arachidonic acid, resulting in reduced prostaglandin synthesis (Fitzgerald & Patrono, 2001). Ibuprofen acts on both COX-1 and COX-2, inhibiting prostaglandins and related compounds located peripherally (Atkinson *et al*., 2015). However, high doses of ibuprofen administration can lead to undesirable adverse effects such as heartburn, gastritis, epigastric pain, and kidney damage, with potential long-term consequences like renal papillary necrosis, peptic ulcers, and gastrointestinal bleeding (Moriarty & Carroll, 2016; Irvine *et al*., 2018).

Therefore, as previously mentioned regarding ibuprofen, all medications carry adverse effects. NSAIDs are known to induce severe side effects, notably impacting the gastrointestinal, renal, and cardiovascular systems (Arfè *et al*., 2016; Ziya Şener & Okşul, 2020; Dreischulte *et al*., 2015; Essex *et al*., 2013). High doses of paracetamol (PCM) have been correlated with hepatotoxicity, kidney and heart problems, and reproductive system toxicity in some studies (Uysal *et al*., 2016; Murad *et al*., 2016; Turtle *et al*., 2013; Aksu *et al*., 2016). Indiscriminate use of dipyrone can lead to severe adversities such as agranulocytosis, interstitial nephritis, alveolitis, pneumonitis, hepatitis, aplastic anemia, Stevens-Johnson syndrome, and toxic epidermal necrosis (Garcez *et al*., 2018; Silva *et al*., 2015).

Moreover, studies have demonstrated that the use of analgesics by adult men can impact testosterone synthesis in the body, resulting in decreased concentration, reduction in germ cell numbers, and induction of oxidative stress, leading to apoptosis of spermatocytes. All these factors can contribute to male infertility (Hurtado-Gonzalez *et al*., 2018; Konkel, 2018).

According to a recent World Health Organization (WHO) report (2023), approximately 1 in 6 individuals (17.5%) globally experience challenges in conceiving. Another significant observation is that infertility affects around 15-20% of reproductive-age couples, with approximately 85% of cases having an identifiable cause, while the cause remains unknown in 15% of cases (Sihag *et al*., 2018; Carson & Kallen, 2021). The male factor can contribute up to 60% to a couple’s infertility but is solely responsible in 20% of cases (Patel *et al*., 2017).

Male infertility is a complex syndrome encompassing various disorders and can result from anatomical, genetic, systemic, neurological, infectious, and immunological factors, among others. Despite advances in understanding male infertility, approximately 50% of cases remain idiopathic, lacking an apparent cause (Poongothai *et al*., 2009).

In recent years, exposure to chemicals and drugs has been associated with male infertility. Agarwal *et al*. (2021) delineated key aspects of male infertility, including primary screening tests, diagnosis, causes, and risk factors. They highlighted exposure to gonad toxicants, including certain medications such as painkillers, chemotherapy drugs, radiotherapy treatments, and contact with pesticides and heavy metals, as factors that should be considered during infertility evaluation. This exposure has been linked to reduced sperm levels in some cases.

In the literature, there is a growing discussion regarding the potential harmful effects on fertility from the prolonged use of non-opioid analgesics. Hence, this study was conceived to establish a correlation between the use of major analgesics and male infertility.

## MATERIALS AND METHODS

### Study Type

This study represents a systematic literature review conducted in adherence to the Preferred Reporting Items for Systematic Reviews and Meta-Analyses (PRISMA statement, 2022) guideline, ensuring comprehensive and rigorous review methodology (Moher *et al*., 2009).

### Search Strategy

To conduct the searches in this study, Medical Subjects Headings (MeSH) were initially reviewed to identify appropriate descriptors. Subsequently, these descriptors were combined using Boolean operators to formulate four distinct searches: (1) Male infertility OR Semen Quality AND Aspirin, (2) Male infertility OR Semen Quality AND Ibuprofen, (3) Male infertility OR Semen Quality AND Acetaminophen, (4) Male infertility OR Semen Quality AND Dipyrone. These search strategies were implemented across three databases: PubMed, SciELO, and LILACS.

### Study Eligibility Criteria

The studies evaluated in this review were those published between June 2013 and March 2023, and they were considered regardless of the language of publication, encompassing articles in English, Portuguese, and Spanish.

### Criteria for Selecting Studies

#### Inclusion Criteria

- In selecting studies for this review, the following eligibility criteria were adopted:
- Cohort studies
- Cross-sectional studies
- Clinical trials
- Complete studies
- Studies with animal models
- Studies that specifically addressed the theme proposed by the authors

#### Exclusion Criteria

The following criteria were used to exclude studies:

Studies falling outside the scope of the research

Review articles

Letters to the editor

Case reports

Duplicate articles

Incomplete articles

Unavailable articles

### Data Collection

Following the execution of the searches, the articles underwent evaluation based on their title and abstract. Those that did not meet the specified criteria were excluded from further consideration. Articles that aligned with the predetermined review criteria were then evaluated by a panel of three authors (M.C.F., M.B.A., and M.G.A.). In cases where unanimous agreement was reached regarding the clinical outcome, the relevant data were tabulated. However, in instances of any discrepancies, a fourth specialist author in the field (M.P.B.) conducted a re-analysis.

### Data Extraction

Upon completing the initial selection process, the chosen articles were carefully reviewed to extract the following data:

- Author’s name
- Year of publication
- Study population
- Number of patients
- Analgesic used
- Administration time
- Dose
- Effect on male fertility

## RESULTS

**Figure 1:**
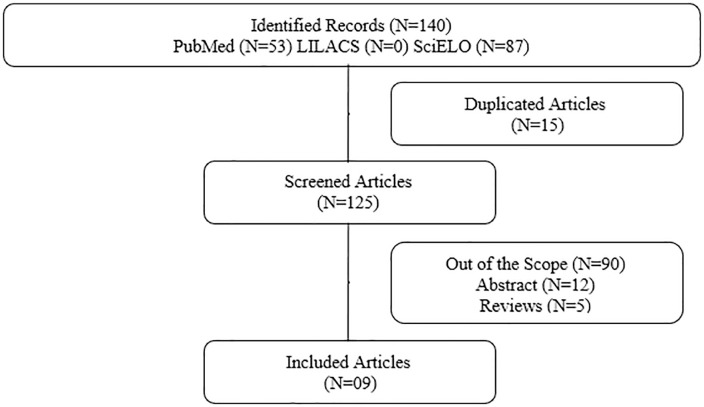
Methodological Screening.

The initial data search yielded a total of 140 articles across PubMed (n=53), SciELO (n=87), and LILACS (n=0). Following the removal of duplicate articles (n=10), 130 unique articles remained for screening. After a thorough review of titles and abstracts, 112 articles that did not meet the inclusion criteria were excluded, leaving 18 articles for full reading. After a comprehensive review of these 18 articles, 9 were selected for detailed analysis (Figure 1).

### Effects of Paracetamol on Male Fertility

Among the 9 selected articles, four investigated the correlation between sperm quality and the use of paracetamol, as summarized in Table 1. Two of these studies utilized C57BL/6 mice, one involved 468 male participants, and one utilized testicular cells for the tests (Albert *et al*., 2013; Holm *et al*., 2015; Smarr *et al*., 2017; Ma *et al*., 2018).

**Table 1:** Changes in male fertility caused by analgesics.

Author	Year	Participant	Number	Analgesic	Dose	Effect
Albert *et al.*	2013	Testicular Cell Culture	12 plates	Paracetamol; Aspirin.	-	↓ testosterone secretion ↓ PGE_2_ and PGD_2_ production
Holm *et al*.	2015	Mouse (C57BL/6 JBomTac)	4 groups of 5 animals	Paracetamol	50 mg/kg	↓ testosterone secretion ↑ the levels of pregnenolone ↓ the anogenital distance
Smarr *et al.*	2017	Men	468 men	Paracetamol	-	↓ sperm volume ↓ motility ↓ morphologically normal spermatozoa ↑ DNA fragmentation
Ma *et al.*	2018	Mouse (C57BL/6)	6 groups of 6 animals	Paracetamol	50 mg/kg; 100 mg/kg; 200 mg/kg; 300 mg/kg.	↑ oxidative stress ↑ amount of dead spermatozoa ↓ motility ↓ sperm density ↓ sperm density
Roodbari *et al.*	2015	Mouse NMRI	3 groups of 12 animals	Ibuprofen	30 mg/kg; 57 mg/kg.	↑ sperm with immature chromatin ↓ sperm count ↓ motility ↓ feasibility
Rey-Ares *et al.*	2018	Human Cells	12 men in regular spermatogenesis	Ibuprofen	10^-4^ M; 10^-6^ M.	↓ PGE_2_ levels ↓ mRNA
Barbosa *et al.*	2020	Wistar Rats	4 groups of 12 animals	Ibuprofen	2.4mg/kg; 7.2mg/kg; 14.3mg/kg.	↓ motility ↓ daily sperm production ↓ sperm quantity ↓ spermatozoa in epididymis
Banihani & Shatnawi	2020	Men	43 samples	Aspirin	0.1 mM; 1.0 mM.	↓ motility ↓ vitality
Martins *et al.*	2021	Wistar Rats	3 groups of 7 animals	Aspirin; Dipyrone.	100 mg/kg	↓ testosterone secretion ↓ daily sperm production ↓ number of spermatids

Caption: ↓ = Reduction; ↑ = Increase; PGE_2_ = Prostaglandin E_2_; PGD_2_ = Prostaglandin D_2_; mRNA = Messenger RNA.

In animal model studies, particularly with C57BL/6 mice, a reduction in testosterone secretion, anogenital distance, sperm motility and density, spermatid density, an increase in pregnenolone levels, oxidative stress, and the number of dead sperm were observed (Holm *et al*., 2015; Ma *et al*., 2018). Another study using testicular cell culture plates demonstrated a decrease in testosterone levels accompanied by a reduction in prostaglandins E2 (PGE2) and prostaglandins D2 (PGD2) (Albert *et al*., 2013). Additionally, a study involving 468 male patients of reproductive age revealed an association between an increase in urinary paracetamol concentration and a reduction in sperm volume, sperm motility, morphometry, and a slight increase in DNA fragmentation (Smarr *et al*., 2017).

### Effects of Ibuprofen on Male Fertility

Three of the articles investigated the association between ibuprofen and male fertility. Two of these studies were conducted using animal models, involving adult NMRI mice and Wistar rats, respectively, while the third study used human cells from 12 men with normal spermatogenesis.

In the study of Roodbari *et al*. (2015) three groups of 12 adult NMRI mice each were utilized, with divisions into a control group, a normal dosage group of 30 mg/kg, and a high dosage group of 57 mg/kg. The study observed a reduction in sperm quality in animals treated with ibuprofen. The study by Barbosa *et al*. (2020) used Wistar rats to examine the effects of ibuprofen on the male reproductive system. A total of 48 animals were equally divided into a control group and three experimental groups receiving different doses of ibuprofen (2.4 mg/kg, 7.2 mg/kg, and 14.3 mg/kg). This research demonstrated a reduction in normal sperm, alterations in sperm tail morphology, decreased motile sperm, reduced daily sperm production, and a decrease in sperm located in the epididymis across all doses of ibuprofen administered in Wistar rats.

In an analysis utilizing human testicular peritubular cells to investigate the effects of ibuprofen, tests were conducted with the drug and prostaglandin E2 (PGE2) in serum-free media for 24 hours. The concentration of ibuprofen used was based on the levels of this analgesic found in the blood of the 12 patients. The tests correlated the use of ibuprofen with a reduction in prostaglandin E2 levels, glial cell line-derived neurotrophic factor (GDNF) levels, and mRNA concentrations (Rey-Ares *et al*., 2018).

### Effects of Aspirin on Male Fertility

Two articles were included in this review to investigate the impact of aspirin (acetylsalicylic acid - ASS) on male fertility. The study by Banihani & Shatnawi (2020) utilized seminal samples from 43 healthy and fertile men. These samples were categorized into three groups: the first exposed to aspirin at a concentration of 0.1mM, the second at 1mM, and the third serving as the control group. Following aliquot preparation, they were incubated for an hour at 37°C, and subsequently examined. The analysis of motility revealed a reduction in both aspirin-exposed groups. Specifically, the group with the highest aspirin concentration exhibited a 43.5% reduction in motility, while the group with the lowest concentration displayed a 16.6% reduction compared to the control group. Sample vitality was also assessed, demonstrating a 16.01% reduction in group 1 and a 3.63% reduction in group 2 relative to the control group. Additionally, two other compounds were quantified in the samples: calcium and nitrite. Both exhibited a reduction, though the reduction in nitrite levels did not reach statistical significance.

In the study of Martins *et al*. (2021), conducted on Wistar rats, the effects of aspirin (AAS) and dipyrone on seminal quality were examined. The aspirin group comprised 7 rats, administered a dose of 100 mg/kg of the drug over a 15-day period. Animals treated with aspirin showed a nearly 60% reduction in testosterone levels, a 56.58% decrease in daily sperm production, and a 56.64% reduction in the number of spermatids when compared to the control group.

### Effects of Dipyrone on Male Fertility

A singular study concerning the adverse effects of dipyrone on male fertility was identified. Martins *et al*. (2021) adopted a methodology similar to the tests conducted with aspirin (AAS). Animals subjected to this medication exhibited a 60% reduction in testosterone levels, a 55.04% decrease in daily sperm production, and a 54.42% reduction in the number of spermatids compared to the control group.

## DISCUSSION

Infertility afflicts more than 180 million individuals globally, with male factors solely contributing to 20% of cases and playing a significant role in 50% of couples’ infertility (Esteves *et al*., 2021). Male infertility stems from diverse causes, ranging from genetic mutations and diseases to lifestyle choices and medication use. Indeed, the administration of analgesics has been associated with risks that can adversely affect reproduction in animals (Agarwal *et al*., 2020; Ratnasooriya & Jayakody, 2000; Høyer *et al*., 2017). Epidemiological studies have revealed that exposure to paracetamol (PCM), either alone or in combination with nonsteroidal anti-inflammatory drugs (NSAIDs), during the first and second trimesters of pregnancy is linked to a higher rate of cryptorchidism (Berkowitz & Lapinski, 1996; Kristensen *et al*., 2016; Stukenborg *et al*., 2021). Jensen *et al*. (2010) and Kristensen *et al*. (2011a) were able to associate the use of analgesics with the incidence of cryptorchidism in newborns. Consequently, literature has suggested that PCM causes endocrine dysregulation and may interfere with the endocrine system in humans, affecting prostaglandin synthesis and testosterone production in men (Wiger *et al*., 1995; Axelstad et al., 2018; Kristensen *et al*., 2011b).

In the study conducted by Mazaud-Guittot *et al*. (2013), the impact of paracetamol, aspirin, and indomethacin on endocrine disruptions in the human fetal testis was investigated, with potential interference in the process of testicular descent. The study explored gene expression and hormone production involved in testicular development in cultures of human fetal testis cells exposed to these drugs. Results demonstrated that paracetamol led to a decrease in testosterone production, while aspirin and indomethacin stimulated its production. All three drugs also caused a decrease in prostaglandin E2 (PGE2) levels in the testicles. Thus, this study highlights that analgesics can induce endocrine disturbances in the human fetal testicle, affecting hormone production and potentially interfering with testicular descent. The approach employed in this study allows for the investigation of the direct effects of these drugs on testicular cells, offering an initial understanding of potential mechanisms involved. However, it is important to acknowledge that full replication of the conditions and complexities of the in vivo environment is not possible, limiting the generalizability of the results to living humans. These findings align with the articles analyzed in the present study, all of which observed a negative correlation between the use of PCM and hormone production.

Albert *et al*. (2013) conducted a study utilizing testicular cells to investigate the effect of paracetamol (PCM) on hormonal levels. Their results demonstrated that PCM possesses the capability to disrupt the production of testosterone in Leydig cells. This reduction in hormone secretion directly impacts seminal quality. Additionally, the study observed that the analgesic exhibits antagonistic activity in the production of prostaglandin E2 (PGE2) and prostaglandin D2 (PGD2). Subsequently, Holm *et al*. (2015) conducted an in vivo study using C57BL/6JBomTac lineage mice. One of the primary objectives was to investigate the connection between exposure to aniline and PCM in the womb and potential effects on the development of the male reproductive system. After histological and endocrine evaluations, it was observed that intrauterine exposure to paracetamol in relevant pharmaceutical doses led to a decrease in testosterone secretion. Furthermore, it resulted in a shortening of the anogenital distance in mice, a sensitive marker of fetal androgen levels. In humans, this marker is associated with reproductive malformations and reproductive disorders in adulthood.

The subsequent year, Holm *et al*. (2015) replicated the study from the previous year, this time analyzing the effects of PCM and aniline on the female reproductive system. The study unveiled that intrauterine exposure is linked to adverse effects on the reproductive development of females. These chemical substances induced alterations in the growth of primary and secondary follicles, consequently decreasing follicle reserves. These structures are vital for egg production and the normal functioning of ovaries. Furthermore, it was observed that exposure could compromise fertility, affecting the ability to conceive and reproduce. Hence, based on these studies, it is plausible to assume that the use of PCM during pregnancy may pose a significant risk to the reproductive health of the offspring, irrespective of gender.

Hormonal alterations were also evidenced in the in vivo study conducted by van den Driesche *et al*. (2015). In this study, two tests were performed: firstly, a xenograft model utilized human fetal testicular tissue in mice to evaluate the effects of administering recommended doses of paracetamol (PCM) for seven days. The results indicated a significant decrease in testosterone levels in the plasma and seminal vesicle weight. Subsequently, they evaluated the impact of intrauterine exposure to PCM on male fertility, revealing that PCM leads to a reduction in testosterone levels by interfering with the expression of critical steroidogenic enzymes. These findings affirm the anti-gonadotropic effect of the studied analgesic on men. Nevertheless, further research endeavors aim to correlate the use of analgesics, such as PCM, with seminal quality issues.

Since 1990, studies have endeavored to establish a relationship between PCM usage, seminal quality, and its effects on male fertility. However, the findings have presented discrepancies (Gandy *et al*., 1990; Ratnasooriya & Jayakody, 2000; Ekaluo *et al*., 2009; Aksu *et al*., 2016). Notably, two articles identified in this review provided evidence supporting the deleterious effects of this medication on seminal quality (Smarr *et al*., 2017; Ma *et al*., 2018). Particularly, the study by Smarr *et al*. (2017), which analyzed 468 men, revealed that participants with elevated urinary PCM levels exhibited alterations in motility, morphology, DNA fragmentation levels, and an increased time to pregnancy. Furthermore, Rossitto *et al*. (2019) demonstrated, through an in utero study with rats, the repercussions of using PCM and ibuprofen during pregnancy. Male offspring exposed to these medications exhibited a 19% reduction in sperm count in semen. The second generation also displayed a 40% reduction in motility when both parents were exposed. Thus, once again, studies underscore the relationship between PCM usage and male fertility. However, comprehensive human studies are imperative to provide a more nuanced understanding.

In contrast to PCM, where research has confirmed its endocrine-disrupting activity in the developing male fetus and its impact on adult male fertility, studies focusing on nonsteroidal anti-inflammatory drugs (NSAIDs) remain inconclusive due to the dearth of research in humans (Drobnis & Nangia, 2017). This class of medications ranks among the most widely used globally, and numerous studies in recent decades have investigated their effects when administered during pregnancy and adulthood (Dean & Sharpe, 2013; Lind *et al*., 2017; Uzun *et al*., 2015; Bagoji *et al*., 2017). In this review, four studies examining the effects of ibuprofen were identified, and their results are consistent, revealing the potential of this class to induce detrimental effects on male fertility (Roodbari *et al*., 2015; Rey-Ares *et al*., 2018; Barbosa *et al*., 2020). The study by Roodbari *et al*. (2015) observed alterations in seminal quality parameters, such as reduced viability, sperm quantity, motility, and decreased chromatin integrity. These findings align with the results reported by Barbosa *et al*. (2020), who also administered ibuprofen in an animal model. In addition to these similar effects, they noted a reduction in the number of sperm in the epididymis and daily production. Another study aimed to evaluate the deleterious effects on spermatogenesis while demonstrating the potential role of selenium in mitigating testicular toxicity induced by ibuprofen. It was observed that the administration of these NSAIDs to rats resulted in decreased volume, global count, and motility. These changes were correlated with an increase in testicular reactive oxygen species (Sharma *et al*., 2020).

Moreover, the impact of ibuprofen use during pregnancy was investigated by Ben Maamar *et al*. (2017), revealing that this analgesic can alter the growth of the male fetal testicle in humans, potentially affecting male reproductive capabilities. In summary, existing literature primarily comprises in vivo studies demonstrating that this anti-inflammatory agent can diminish sperm quality, with documented reductions in motility, viability, sperm count, and DNA integrity (Stutz *et al*., 2000; Robinson *et al*., 2005; Ingram *et al*., 2006; Rocco *et al*., 2012).

Despite the substantial research on the effects of nonsteroidal anti-inflammatory drugs (NSAIDs) on male fertility, scant research is available in databases analyzing the impacts of acetylsalicylic acid (ASA) on seminal quality. Hence, only three articles were uncovered in this review that correlate the use of the drug with its anti-androgenic properties and detrimental effects on male fertility (Albert *et al*., 2013; Banihani & Shatnawi, 2020; Martins *et al*., 2021). Exposure to ASA in human renal cortex cells at concentrations of 0.01 and 0.1mM resulted in a notable reduction in testosterone levels of approximately 18% and 17%, respectively, over 24 hours, and approximately 14% and 12%, respectively, over 48 hours (Albert *et al*., 2013). Additionally, a study employing rat fetal testes concluded that aspirin exerts specific and direct antiandrogenic effects (Kristensen *et al*., 2012). In a study conducted in India in 2018, an in vivo analysis utilizing rats found that aspirin usage over one to two months led to a reduction in motility, density, and total quantity of sperm in the semen (Vyas *et al*., 2016). Furthermore, assessments with 43 human seminal samples indicated reduced motility and vitality following exposure to ASA at concentrations of 0.1 and 1.0mM for one hour (Banihani & Shatnawi, 2020). Consequently, there is a consensus among researchers that ASA has an adverse impact on semen quality parameters.

In humans, cyclooxygenase (COX) inhibitors such as ASA, PCM, or ibuprofen have been associated with compromised sperm quality. Nonetheless, in-depth investigations regarding the effects of dipyrone on the male reproductive system and the involvement of the epididymis in the fertility-impeding effects of COX inhibitors are warranted (Drobnis & Nangia, 2017; Kristensen *et al*., 2012; Sadeghzadeh *et al*., 2019). Moreover, there is a paucity of studies in the existing literature concerning this topic, largely due to the prohibition of the sale and medical use of this medication in key research-centric countries such as the United Kingdom, Canada, and the United States owing to its adverse effects (de Leeuw *et al*., 2017; Nikolova *et al*., 2012; Siramshetty *et al*., 2016).

The sole article identified in our research reported that the daily administration of 100 mg/kg of dipyrone for 15 days in Wistar rats resulted in decreased sperm production, sperm count in the epididymis, and serum testosterone levels (Martins *et al*., 2021). However, the precise mechanism underlying the reduction of testosterone levels in the body induced by non-opioid analgesics and NSAIDs remains uncertain. Consequently, a study conducted in Brazil combined in vivo and in vitro tests to scrutinize the effects of dipyrone on steroidogenesis and its potential receptor-mediated anti-androgenic effects. The findings revealed a reduction in testosterone production, attributed to the inhibition of enzymes at various stages of steroidogenesis. Nonetheless, it was concluded that dipyrone did not exert an anti-androgenic effect on the body (Passoni *et al*., 2018).

## CONCLUSION

The literature emphasizes the detrimental effects of paracetamol and ibuprofen on male fertility, substantiated by comprehensive in vitro and in vivo investigations. However, a significant gap exists in clinical research, warranting further exploration into optimal administration timing and dosage. The impact of aspirin and dipyrone on male fertility remains ambiguous due to a scarcity of studies and disparate methodologies employed. Adequate in vitro and in vivo research is imperative to comprehensively understand the effects of aspirin. Moreover, the dearth of studies exploring the influence of dipyrone on male fertility poses a challenge in establishing a concrete correlation, underscoring the necessity for additional research in this domain.

## List of Abbreviations

Aspirin (AAS)

Nonsteroidal anti-inflammatory drugs (NSAIDs)

Cyclooxygenase (COX)

Paracetamol (PCM)

Prostaglandin D2 (PGD2)

Prostaglandin E2 (PGE2)
